# TNF-α (G-308A) Polymorphism, Circulating Levels of TNF-α and IGF-1: Risk Factors for Ischemic Stroke—An Updated Meta-Analysis

**DOI:** 10.3389/fnagi.2022.831910

**Published:** 2022-03-16

**Authors:** Ranran Duan, Na Wang, Yanan Shang, Hengfen Li, Qian Liu, Li Li, Xiaofeng Zhao

**Affiliations:** ^1^Department of Neurology, First Affiliated Hospital of Zhengzhou University, Zhengzhou, China; ^2^Department of Neurorehabilitation, Second Affiliated Hospital of Zhengzhou University, Zhengzhou, China; ^3^Department of Psychiatry, First Affiliated Hospital of Zhengzhou University, Zhengzhou, China; ^4^Department of Anesthesiology, Beijing Friendship Hospital, Capital Medical University, Beijing, China

**Keywords:** TNF-α, IGF-1, ischemic stroke, meta-analysis, gene polymorphism

## Abstract

**Objective:**

Accumulated studies have explored gene polymorphisms and circulating levels of tumor necrosis factor (TNF)-α and insulin-like growth factor (IGF)-1 in the etiology of ischemic stroke (IS). Of the numerous etiopathological factors for IS, a single-nucleotide polymorphism (SNP) rs1800629 located in the *TNF*-α gene promoter region and increased levels of TNF-α were found to be associated with IS in different ethnic backgrounds. However, the published results are inconsistent and inconclusive. The primary objective of this meta-analysis was to investigate the concordance between rs1800629 polymorphism and IS. A secondary aim was to explore circulating levels of TNF-α and IGF-1 with IS in different ethnic backgrounds and different sourced specimens.

**Methods:**

In this study, we examined whether rs1800629 genetic variant and levels of TNF-α and IGF-1 were related to the etiology of IS by performing a meta-analysis. Relevant case-control studies were retrieved by database searching and systematically selected according to established inclusion criteria.

**Results:**

A total of 47 articles were identified that explored the relationship between the rs1800629 polymorphism and levels of TNF-α and IGF-1 with IS risk susceptibility. Statistical analyses revealed a significant association between the rs1800629 polymorphism and levels of TNF-α and IGF-1 with IS pathogenesis.

**Conclusion:**

Our findings demonstrated that the TNF-α rs1800629 polymorphism, the increased levels of TNF-α, and decreased levels of IGF-1 were involved in the etiology of IS.

## Introduction

Ischemic stroke (IS) is the second leading cause of mortality and physical disability following the onset of neuropathological complications worldwide (Strong et al., 2007; Navis et al., 2019). Presently, it is crucial to identify the potential risk factors of IS that are associated with challenges in the early detection of stroke symptoms as well as poor survival outcomes.

Pro-inflammatory cytokines play a major role in the process of activation of various cellular mechanisms, including differentiation and proliferation processes. If the pro-inflammatory cytokine storm occurs in the cerebral tissue (King et al., 2013; Clausen et al., 2014; Arango-Dávila et al., 2015; Wu et al., 2016), it will contribute to additional brain injuries, such as IS (Jiang et al., 2020). Among the identified cytokines, the pathogenic roles of tumor necrosis factor α (TNF-α) have been extensively investigated. It has been demonstrated that an increased level of TNF-α is associated with greater neurological deficits and poorer treatment outcomes in IS patients (Pasarica et al., 2005; Cui et al., 2012; Bokhari et al., 2014; Boehme et al., 2016; Lasek-Bal et al., 2019). In preclinical studies, the application of TNF-α agonists or reduced expression of TNF-α has exhibited potential neuroprotective effects in the cerebral cortex of IS patients. Notably, Clausen et al. (2020) have demonstrated significantly increased risks of stroke in patients with elevated concentrations of TNFR1 and TNFR2 factors in plasma, suggesting that TNF-α level might serve as a risk factor for IS as well as the biomarker for the patient survival rate. Similarly, insulin-like growth factor-1 (IGF-1) that demonstrates cell proliferation, an inhibitor of cell apoptosis, exerts neuroprotective effects in both white and gray matter under different detrimental conditions. It is a key regulator of cell proliferation and an inhibitor of cell apoptosis and necrosis a key regulator in the development, cell differentiation, plasticity and survival of the nervous system (Juul, 2003; Benarroch, 2012; Shaheen et al., 2018). Based on this evidence, it can be speculated that IGF-1 might be involved in the etiology of IS.

Previous studies have also explored the association between the *TNF*-α gene polymorphism and peripheral blood levels of TNF-α and IGF-1 with IS onset in different ethnic-specific backgrounds. However, the findings from such studies have been inconsistent or inconclusive due to their highly diverse patients’ populations, differentially sourced specimens, small sample sizes, and most importantly, little influence of single-nucleotide polymorphisms (SNPs) on IS pathobiology. To overcome these roadblocks, an updated meta-analysis was performed to further testify whether (1) the *TNF*-α genetic SNPs could increase the susceptibility of different populations to IS and (2) the peripheral blood TNF-α and IGF-1 levels could be used as the biomarker for assessing the risks for IS in ethnic-specific background.

## Methods

### Inclusion Criteria

For this meta-analysis, full-length relevant articles and case studies were selected based on the following inclusion criteria: (1) The diagnosis of IS patients was based on the CT or MRI examinations; (2) selected studies were all case-control studies; (3) data on SNP frequency of circulating TNF-α and IGF-1 levels were clearly obtained; and (4) the frequency of each allele and genotype met the criteria for Hardy-Weinberg disequilibrium in both the case and control groups. In contrast, some studies were excluded based on the criteria that they were either case-only studies based on a family design or abstract-only reviews without the full case report.

### Search Strategy

We used the keywords “tumor necrosis factor-alpha,” human recombinant tumor necrosis factor-alpha, “cachectin/tumor necrosis factor,” tumor necrosis factor ligand superfamily member 2, “TNF alpha,” “TNF-alpha insulin-like somatomedin peptide I,” “insulin-like somatomedin peptide I,” “IGF-I-SmC,” “IGF-1 insulin-like growth factor I,” “ischemic stroke,” “cryptogenic stroke,” and “cryptogenic embolism stroke” to search for the full-length case studies in the PubMed, Web of Science, Embase, Elsevier, Cochrane, Medline, and APA databases published until September 9, 2021. We thoroughly reviewed the retrieved literature and the corresponding reference lists. To avoid including duplicate articles, we selected studies from the larger sample size in the analysis.

### Data Extraction

Three of the authors independently extracted the following data from each publication: Author, country of origin, racial descent of the study population, the number of eligible cases with proper controls, circulating levels of TNF-α, IGF-1 (mean and standard deviation), rs1800629 (G-308A) genotype, and allele frequencies.

### Data Analysis and Statistical Methods

The data analysis using statistical methods was carried out according to the methods described in our previous study (Zhao et al., 2013).

## Results

**FIGURE 1:**
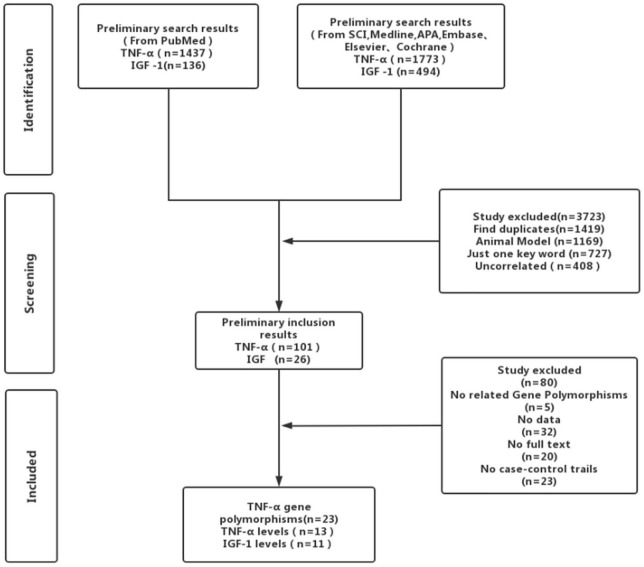
Flowchart of the included articles.

Strictly following the inclusion criteria, 47 properly controlled full-length studies, including 23 rs1800629 genotype-related studies (Um et al., 2003; Balding et al., 2004; Lee et al., 2004; Um and Kim, 2004; Karahan et al., 2005; Rubattu et al., 2005; Harcos et al., 2006; Lalouschek et al., 2006; Llamas Sillero et al., 2007; Banerjee et al., 2008; Shi et al., 2009; Kim et al., 2010; Tong et al., 2010; Sultana et al., 2011; Cui et al., 2012; Tuttolomondo et al., 2012; Zhao et al., 2012; Djordjevic et al., 2013; Wawrzynek et al., 2014; Gu et al., 2015; Kumar et al., 2016; Palm et al., 2020; Kamdee et al., 2021) and 13 case reports (Zaremba and Losy, 2001a,b; Intiso et al., 2004; Domac et al., 2007; Jefferis et al., 2009; Cure et al., 2013; Bokhari et al., 2014; de Sousa Parreira et al., 2015; Wytrykowska et al., 2016; Billinger et al., 2017; Shishkova et al., 2018; Zhang Y. Y. et al., 2018; Ma et al., 2020) on the circulating level of TNF-α were finally selected for the meta-analysis (Figure 1). Genotype-related literature included a total of 9,120 patients and 9,249 healthy controls (Caucasian, *n* = 10; Asian, *n* = 15). PCR screening was used to detect genotypes, and all genotypes met Hardy-Weinberg disequilibrium criteria. Similarly, the TNF-α circulation levels were measured in 1,497 cases and 1,444 control subjects. The distribution of articles based on the ethnic specificity was Caucasian (*n* = 6), Asian (*n* = 6), and American (*n* = 2) and that based on the laboratory findings was plasma data (*n* = 3), serum data (*n* = 9), cerebrospinal fluid (CSF) data (*n* = 1), and gingival fluid data (*n* = 1). We identified 11 articles concerning the relationship between TNF-α circulation levels and IS, three articles for plasma data (Schwab et al., 1997; Kaplan et al., 2007; Wang Y. et al., 2014), eight articles for serum data (Denti et al., 2004; Johnsen et al., 2005; Aberg et al., 2011; Iso et al., 2012; Dong et al., 2014; Wang T. et al., 2014; Shaheen et al., 2018; Zhang W. et al., 2018); 2,075 patients in case group; and 2,174 in the control group (Tables 1–3). All the selected articles were analyzed using the STATA15.0 software.

**TABLE 1:** Summary of studies exploring the relationship between rs1800629 of TNF-α gene polymorphism and IS.

References	Race	Case			Control			Case		Control	
					
		GG	GA	AA	GG	GA	AA	G	A	G	A
Um et al. (2003)	Asian	261	32	1	484	82	15	554	34	1,050	112
Balding et al. (2004)	Caucasian	59	40	6	233	140	16	158	52	606	172
Lee et al. (2004)	Asian	139	13	0	138	25	2	291	13	3.1	29
Um and Kim (2004)	Asian	325	40	1	509	86	15	690	42	1,104	116
Karahan et al. (2005)	Caucasian	70	16	0	15	8	0	156	16	158	8
Rubattu et al. (2005)	Caucasian	84	29	2	152	27	1	197	33	331	29
Harcos et al. (2006)	Caucasian	262	71	3	229	97	7	595	77	555	111
Lalouschek et al. (2006)	Caucasian	282	116	6	310	97	8	680	128	717	113
Sillero et al. (2007)	Caucasian	248	42	2	245	51	6	538	46	541	63
Banerjee et al. (2008)	Asian	150	25	1	181	31	0	325	27	393	31
Shi et al. (2009)	Asian	63	4	0	64	6	0	130	4	134	6
Kim et al. (2010)	Asian	220	17	0	190	25	1	457	17	405	27
Tong et al. (2010)	Asian	597	51	0	552	96	0	1,245	51	1,200	96
Tong et al. (2010)	Asian	92	8	0	70	30	0	192	8	170	30
Sultana et al. (2011)	Asian	17	211	10	12	207	7	245	231	221	224
Cui et al. (2012)	Asian	1,237	148	3	886	136	5	2,622	154	1,908	146
Cui et al. (2012)	Asian	868	90	3	710	107	4	1,826	96	1,527	115
Tuttolomondo et al. (2012)	Caucasian	83	12	1	35	11	2	178	14	81	15
Zhao et al. (2012)	Asian	993	127	4	1,028	131	4	2,113	135	2,187	139
Djordjevic et al. (2013)	Caucasian	21	5	0	69	28	3	47	5	166	34
Wawrzynek et al. (2014)	Caucasian	87	9	5	84	15	1	181	19	183	17
Gu et al. (2015)	Asian	508	100	8	501	99	7	1,116	116	1,101	113
Kumar et al. (2016)	Asian	218	29	3	225	23	2	465	35	473	27
Palm et al. (2020)	Caucasian	567	209	17	322	124	11	1,343	243	758	148
Kamdee et al. (2021)	Asian	166	31	3	181	19	0	363	37	381	19
											

**TABLE 2:** Summary of studies exploring the relationship between the levels of TNF-α and IS.

References	Case		Control		Sample size		Origin
			
	Mean	*SD*	Mean	*SD*	Case	Control	
Bokhari et al. (2014)	64.00	68.80	1.00	2.10	131	47	Plasma
de Sousa Parreira et al. (2015)	7.70	6.44	9.20	9.00	93	134	Plasma
Billinger et al. (2017)	2.30	7.00	2.00	6.50	12	14	Plasma
Intiso et al. (2004)	30.10	12.50	29.00	13.90	41	40	Serum
Zaremba and Losy (2001b)	14.00	10.20	9.10	1.60	23	15	Serum
Domac et al. (2007)	44.32	16.92	16.70	5.50	70	22	Serum
Jefferis et al. (2009)	1.83	0.79	1.81	0.77	299	587	Serum
Cure et al. (2013)	75.60	25.00	65.90	9.10	54	50	Serum
Wytrykowska et al. (2016)	12.86	7.33	10.26	4.38	42	34	Serum
Shishkova et al. (2018)	1.29	1.00	1.14	1.57	196	119	Serum
Zhang W. et al. (2018)	3.88	2.30	2.53	1.55	182	40	Serum
Ma et al. (2020)	118.50	27.20	96.20	23.60	288	300	Serum
Zaremba and Losy (2001a)	9.10	5.80	6.60	0.50	23	15	Cerebrospinal fluid
Wytrykowska et al. (2016)	74.78	108.89	30.31	51.37	43	27	Gingival crevicular fluid

**TABLE 3:** Summary of studies exploring the relationship between the levels of IGF-1 and IS.

References	Race	Case		Control		Sample size		Origin
				
		Mean	*SD*	Mean	*SD*	Case	Control	
Schwab et al. (1997)	No mentioned	105.00	23.00	161.00	10.00	20	8	Plasma
Kaplan et al. (2007)	African-American	150.30	58.30	152.20	58.30	370	1,122	Plasma
Wang Y. et al. (2014)	China	122.32	86.60	77.60	53.15	79	75	Plasma
Denti et al. (2004)	Italy	69.00	45.00	102.00	67.00	85	88	Serum
Johnsen et al. (2005)	Danish	107.10	33.30	112.90	33.20	254	254	Serum
Aberg et al. (2011)	Western Sweden	171.00	3.30	145.40	9.40	407	40	Serum
Iso et al. (2012)	Japan	113.00	52.00	106.00	48.00	71	71	Serum
Dong et al. (2014)	China	130.40	32.80	141.40	25.40	240	200	Serum
Wang T. et al. (2014)	China	107.16	18.03	122.79	15.55	124	96	Serum
Shaheen et al. (2018)	Arab	143.40	12.60	156.00	11.20	200	100	Serum
Zhang W. et al. (2018)	China	115.50	37.30	158.50	27.80	225	120	Serum

**FIGURE 2:**
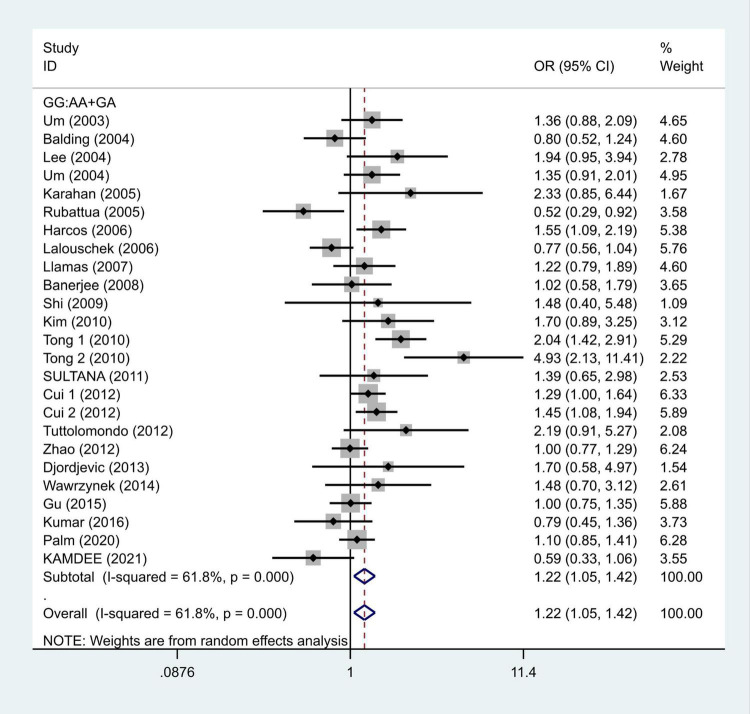
Results of the random-effects meta-analysis for the TNF-α G308A genotype (GG vs. AA + GA) in IS and control groups.

**FIGURE 3:**
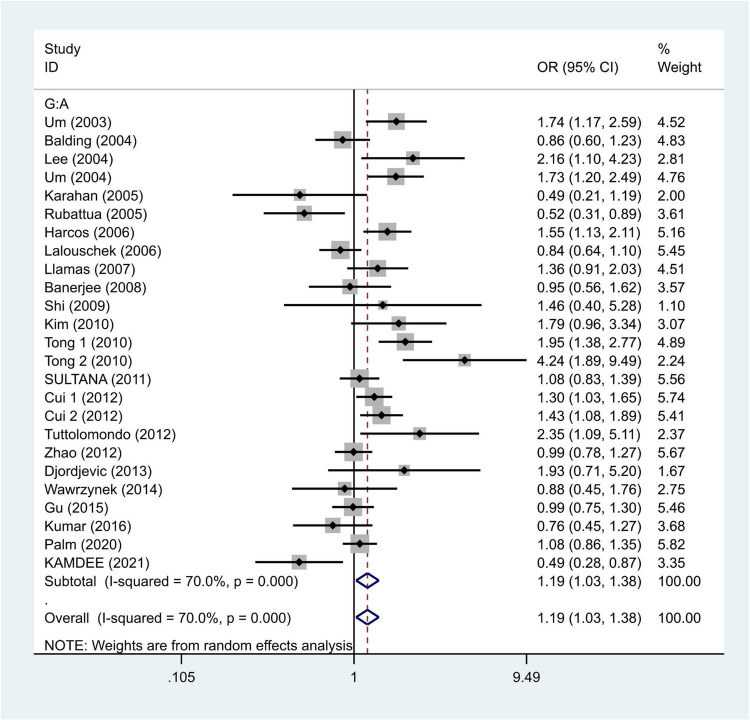
Results of the random-effects meta-analysis for the TNF-α G308A allele (G vs. A) in IS and control groups.

We selected the nucleotide G to A modification as the risk factor among genotypes and established 2 models (GG vs. GA + AA and G vs. A). The heterogeneity test in each group indicated the presence of high heterogeneity among the samples. Heterogeneity models I–V were randomly selected for testing, which showed results for each model, namely, GG vs. GA + AA model [odds ratio (OR) = 1.22, 95% confidence interval (CI) = 1.05–1.42, Figure 2] and G vs. A model (OR = 1.19, 95% CI = 1.03–1.38, Figure 3). We found positive correlations for the GG vs. GA + AA and G vs. A models, suggesting that G-A mutation might be a risk factor for IS.

**FIGURE 4:**
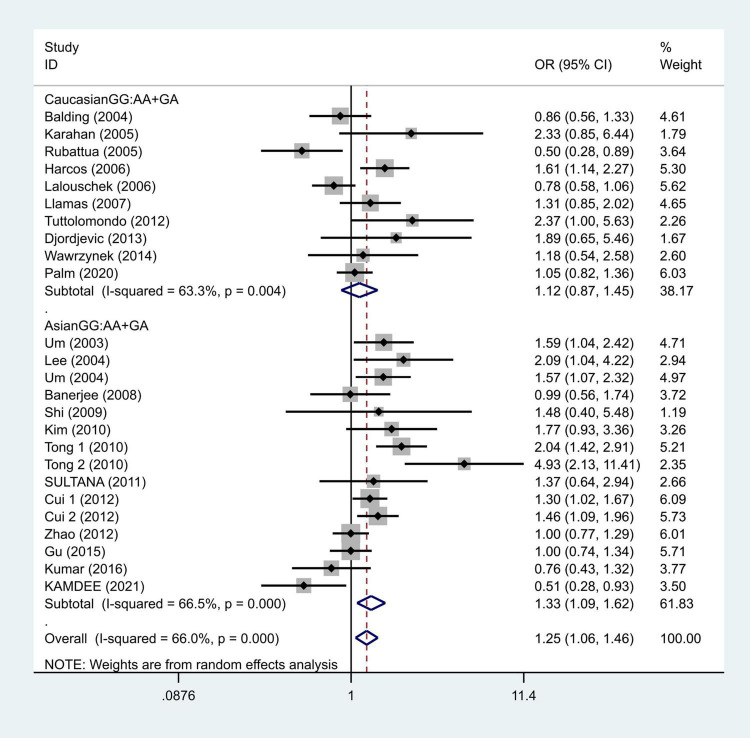
Results of the random-effects meta-analysis for the TNF-α G308A genotype (GG vs. AA + GA) in Caucasian, Asian, IS, and control groups, respectively.

**FIGURE 5:**
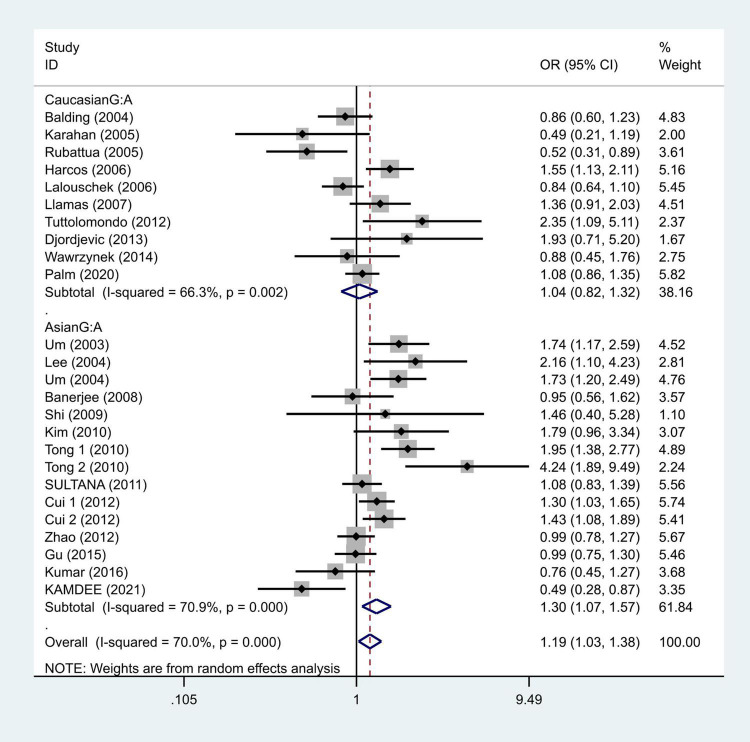
Results of the random-effects meta-analysis for the TNF-α G308A allele (G vs. A) in Caucasian, Asian, IS, and control groups, respectively.

To further study the source of heterogeneity and whether there were differences among races, we conducted a racial stratification analysis on the same dataset. Among them, positive results were found in the Asian race with the models such as GG vs. GA + AA (OR = 1.33, 95% CI = 1.09–1.62, Figure 4) and G vs. A (OR = 1.30, 95% CI = 1.07–1.57, Figure 5), while no positive results were found in case of the Caucasian population. Together, these results indicate that the intensity of G modification-associated risk factors can be linked to racial differences, especially as a potential risk factor for the Asian population.

In the TNF-α level detection, the data of Q25–Q75 in the original text were transformed into mean and SD. We conducted continuous variable analysis by recording mean, SD, and total number.

**FIGURE 6:**
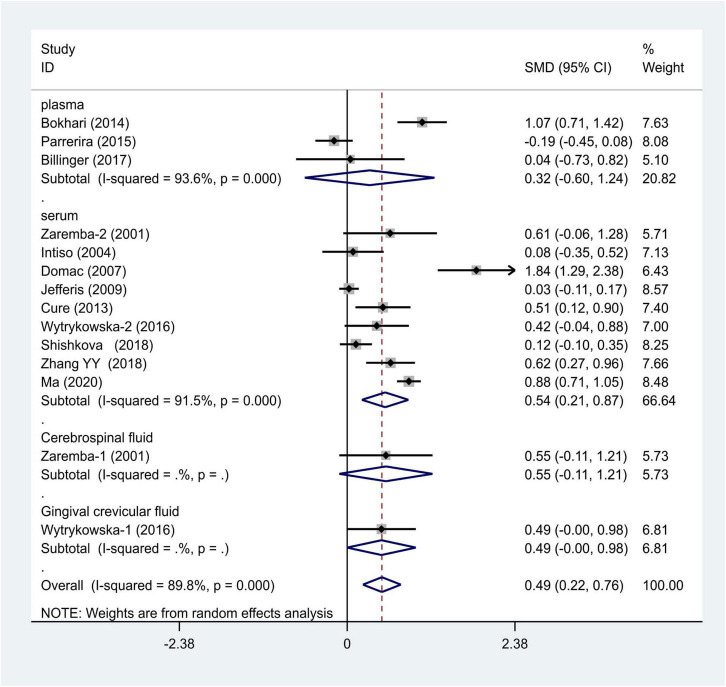
Results of meta-analysis for the level of TNF-α and IS.

**FIGURE 7:**
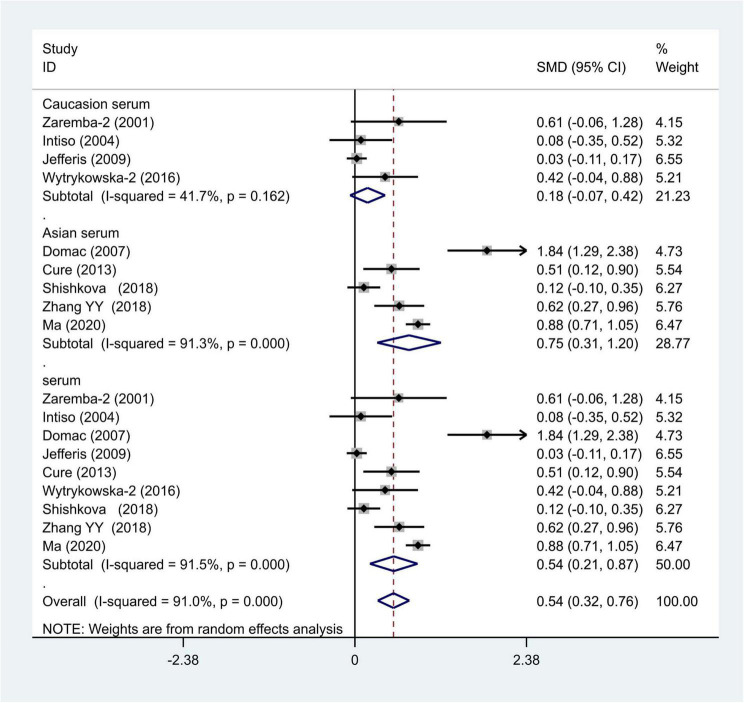
Results of meta-analysis for the level of TNF-α in the Asian and Caucasian populations, respectively.

**FIGURE 8:**
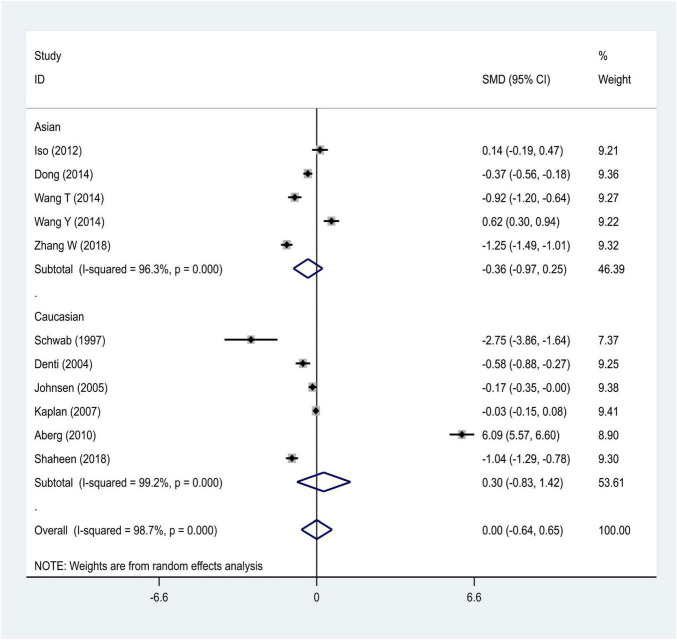
Results of meta-analysis for the level of IGF-1 in the Asian and Caucasian populations, respectively.

**FIGURE 9:**
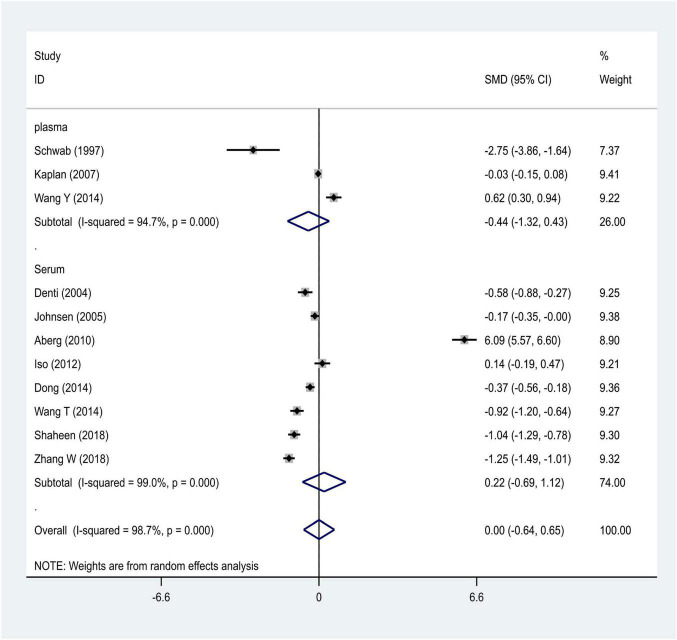
Results of meta-analysis for the IGF-1 level in plasma and serum, respectively.

**FIGURE 10:**
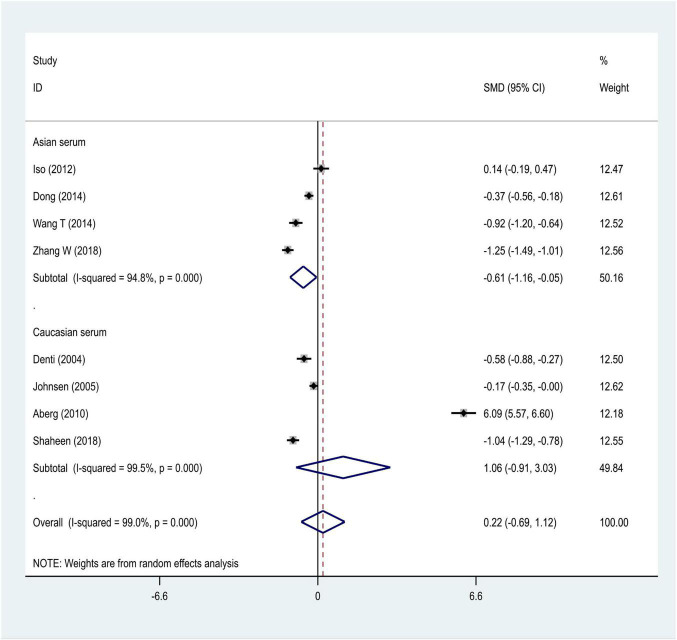
Results of meta-analysis for the IGF-1 serum levels in Caucasian, Asian, IS, and control groups, respectively.

We distributed the cases into the particular group to establish the model based on the sample source. We found that the serum model had a positive result with the SMD value of 0.54 (95% CI = 0.21–0.87, Figure 6) and also had heterogeneity in sample quality. We need to conduct further studies to identify if the advent of heterogeneity can be caused by racial differences. Furthermore, non-positive results were found in the South American population, and positive results were found in the Asian (SMD = 0.75, 95% CI = 0.31–1.20, Figure 7) and Caucasian populations (SMD = 0.18, 95% CI = –0.07–0.42, Figure 7). To further explore the interactions between serological indicators and races, serum results were grouped according to ethnic specificity, which yielded positive results for the Asian population (SMD = 0.75, 95% CI = 0.31–1.20, Figure 7). Therefore, elevated serum TNF-α levels were considered to be a risk factor for IS in the Asian population. The same method was conducted to explore the connection of IGF-1 concentration with IS in different ethnic backgrounds. The decreased serum IGF-1 levels were considered to be a risk factor for IS in the Asian population not in the Caucasian population (SMD = –0.61, 95% CI = –1.16 to –0.50, Figures 8–10).

### Sensitivity Analysis

Sensitivity analysis of the data showed that it did not affect the results of meta-analysis (OR = 1.17, 95% CI = 1.09–1.26 for rs1800629; OR = 0.54, 95% CI = 0.21–0.87 for TNF-α; OR = –0.61, 95% CI = –1.16 to –0.05 for IGF-1).

### Publication Bias

Begg’s test was used to verify publication bias, which showed that publication bias was negligible (*z* = 1.24, *p* = 0.22 for rs1800629; *z* = 0.73, *p* = 0.47 for TNF-α; *z* = 0.96, *p* = 0.34 for IGF-1).

## Discussion

This updated meta-analysis study provided evidence that the rs1800629 SNP was associated with IS susceptibility. In addition, the level of TNF-α cytokine was elevated in IS patients compared with that in controls. Of particular interest, we first explored the relationship between levels of IGF-1 in the etiology of IS by performing a meta-analysis method. Especially, in the subgroup analysis, increased levels of TNF-α cytokine were found among the Asian and Caucasian populations, respectively. In contrast, no significant differences were found between IS patients and controls regarding the concentrations of IGF-1. Notably, we also first explored the interactions between differentially sourced specimens and specific ethnic backgrounds. Based on the stratification of the specimen and specific ethnic background, elevated concentrations of TNF-α and decreased levels of IGF-1 were noted in the serum samples of IS patients in the Asian population but not in the Caucasian population. These findings help us understand the role of gene polymorphisms and abnormal levels of cytokines such as TNF-α and IGF-1 in the pathogenesis of IS. In line with previous findings, our results also demonstrated that the rs1800629 polymorphisms were associated with the IS pathogenesis (Wu et al., 2019). The level of TNF-α was increased in IS patients as compared with that of the control subjects. Importantly, our updated meta-analysis, including 44 studies with 11,480 patents related to genetic SNPs and serum levels of cytokines in IS patients, was larger than the previous study (Wu et al., 2019). We speculated that the greater number of studies and larger sample sizes might obtain precise and valuable results.

The human *TNF*-α gene is located on chromosome 6p21.1–21.3 within the highly polymorphic region of the major histocompatibility complex (MHC). Among the several known *TNF*-α gene polymorphisms, studies have extensively focused on the G-308A (rs1800629) mutation in the etiology of IS. The polymorphisms are reportedly located in the *TNF*-α gene promoter region, which contains a number of regulatory elements that can influence the transcriptional activity of the gene (Wilson et al., 1997), and may also influence the DNA binding affinity of transcription factors leading to differential expressions of the *TNF*-α gene and the disease susceptibility (Wilson et al., 1997). Hence, it has been suggested that a more common regulatory variant may be a more likely risk factor for common disorders than rare variants within the gene coding region (Zill et al., 2002). Notably, the G308A (rs1800629) variant involved SNP mutation in the promoter region, which could upregulate the *TNF*-α gene’s transcriptional activity, resulting in the increased plasma concentrations of TNF-α in IS pathogenesis, and this finding was verified using genetic association analysis.

In IS, the activated microglia and astrocytes release high levels of TNF-α, which is considered toxic for negatively influencing synaptic transmission and plasticity in the learning and memory processes (Beattie et al., 2002), which is the core symptom of IS patients. In contrast, TNF-α combines with its receptors, leading to NF-κB activation that may play dual roles in inducing neurotoxicity as well as neuroprotective responses in brain cells depending on the stage of neuronal development, target cell type, and receptor subtypes (Shohami et al., 1999; Vila et al., 2000; Wang et al., 2000; Castillo and Leira, 2001; Cárdenas et al., 2002; Hallenbeck, 2002; Hurtado et al., 2002). Moreover, in clinical studies, patients with high TNF-α levels (Vila et al., 2000; Wang et al., 2000) have been shown to develop greater neurological deficits and poorer outcomes. In contrast, blocking TNF-α improves clinical outcomes (Tobinick et al., 2012). Similar phenomena have also been observed in several preclinical studies (King et al., 2013; Clausen et al., 2014; Arango-Dávila et al., 2015; Wu et al., 2016; Lin et al., 2020). Importantly, posttreatment with TNF-α neutralizing antibodies or TNF-α agonists alleviates poststroke brain injury in rodents (Vakili et al., 2011). Furthermore, at the molecular level, R-7050 acts as an anti-TNF-α receptor inhibitor demonstrating protection against stroke-induced brain injury (King et al., 2013; Clausen et al., 2014; Arango-Dávila et al., 2015; Wu et al., 2016) as well as enhancing the brain TNFRI and NF-κB signaling cascades along with increased levels of the Nrf2 protein in stroke rats, suggesting that R-7050 may enhance Nrf2 signaling, thus representing the involvement of another signal transduction to alleviate inflammatory responses in IS (Lin et al., 2021). Regarding the role of inflammation in stroke pathogenesis, Della Corte (Della Corte et al., 2016) has reviewed the role of immune-inflammatory variables in patients with IS, assessing the therapeutic perspectives that it offers. Patients with the cardioembolic (CEI) subtype of IS was reported to show significantly higher median plasma levels of TNF-α, compared with that of other subtypes. Multiple linear regression showed a significant association between the Scandinavian Stroke Scale (SSS) score at admission and diagnostic subtype infarct volume of cardioembolic strokes and some inflammatory variable. Tuttolomondo suggested that cardioembolic strokes have a worse clinical presentation and produce larger and more disabling strokes than other IS subtypes reporting a possible explanation of higher immuno-inflammatory activation of the acute phase (Albanese et al., 2005; Tuttolomondo et al., 2008).

Therefore, in stroke, the TNF-α signal transduction is activated during ischemic injuries, and this fact has been further verified in subsequent clinical studies. In our study, the presence of the TNF-α-308 GG genotype and a higher serum concentration of TNF-α increases the likelihood of a stroke pathology. Thus, the TNF-α signal transduction response may explain our results. The molecular mechanism of the association between IGF-1 and IS has not yet been fully elucidated. Previous studies have demonstrated that the IGF-1 couples with protein-3 primarily *via* the PI3-kinase pathway, which, on the one hand, mediates cell survival of neurons under oxidative stress (Gustafsson et al., 2004), and, on the other hand, is preceded by improvement in the blood-brain barrier and suppression of local inflammatory mediators, indicating a unique anti-inflammatory role for IGF-1 in the blood–brain barrier as a novel target for IGF-1-mediated neuroprotection response (Bake et al., 2014; De Geyter et al., 2016). This was verified in both preclinical and clinical studies and our results (Bake et al., 2014; De Geyter et al., 2016; Mehrpour et al., 2016; Lee et al., 2021). This meta-analysis results demonstrated the association between serum IGF-1 levels and ischemic stroke in the Asian population not in the Caucasian population. These differences can be explained by different genetic backgrounds.

One major limitation for this study is that except for different genetic backgrounds, we did not address the relationship between proinflammatory gene polymorphisms and stroke subtypes, because a more common, regulatory variant may be more likely to be involved in the etiology of IS (Hahn and Blakely, 2002). It has been suggested that etiologic factors for the development of the particular subtype may be different in stroke subtypes. As it has been mentioned except for other risk factors, age and sex may also involve in the etiology of different stroke subtypes (Moond et al., 2020; Tang et al., 2020). Thus, in future studies, we will consider stroke severity, subtype, hypertension, dyslipidemia, diabetes, and psychosocial stress in the etiology of IS (Sarfo et al., 2022).

## Conclusion

This updated meta-analysis study demonstrated that the GG genotype might be considered as a risk factor for IS (especially in Asians), and the circulating levels of TNF-α were elevated in the Caucasian and Asian patients as compared with controls. At the same time, a positive association was found between serum IGF-1 levels and IS in the Asian population but not in the Caucasian population. Therefore, based on previous meta-analysis results and those combined with ours, we proposed that therapeutic strategies to decrease the circulating levels of TNF-α and increased levels of IGF-1 might be considered as a promising therapeutic target with potential neuroprotective effects for the treatment of IS.

## Data Availability Statement

The original contributions presented in the study are included in the article/Supplementary Material, further inquiries can be directed to the corresponding author/s.

## Author Contributions

XZ and LL equally contributed to the study design of this review. RD, NW, and YS equally performed the literature search, interpreted the data, and wrote the manuscript. QL and HL profoundly enriched the manuscript by adding important intellectual content. All authors contributed to the article and approved the submitted version.

## Conflict of Interest

The authors declare that the research was conducted in the absence of any commercial or financial relationships that could be construed as a potential conflict of interest.

## Publisher’s Note

All claims expressed in this article are solely those of the authors and do not necessarily represent those of their affiliated organizations, or those of the publisher, the editors and the reviewers. Any product that may be evaluated in this article, or claim that may be made by its manufacturer, is not guaranteed or endorsed by the publisher.

## Supplementary Material

Supplementary Figure 1 The result of publication bias between the allele of TNF-α (G:A) and IS.

Supplementary Figure 2 The result of publication bias between IGF-1, TNF-α, and IS. **(A)** The result of publication bias between IGF-1 and IS and **(B)** the result of publication bias between TNF-α and IS.
