# Supplementary figures and images for: TNF-α (G-308A) Polymorphism, Circulating Levels of TNF-α and IGF-1: Risk Factors for Ischemic Stroke—An Updated Meta-Analysis
Source: Front Aging Neurosci. 2022 Mar 16;14:831910. doi: 10.3389/fnagi.2022.831910 (PMC8966404; doi:10.3389/fnagi.2022.831910)

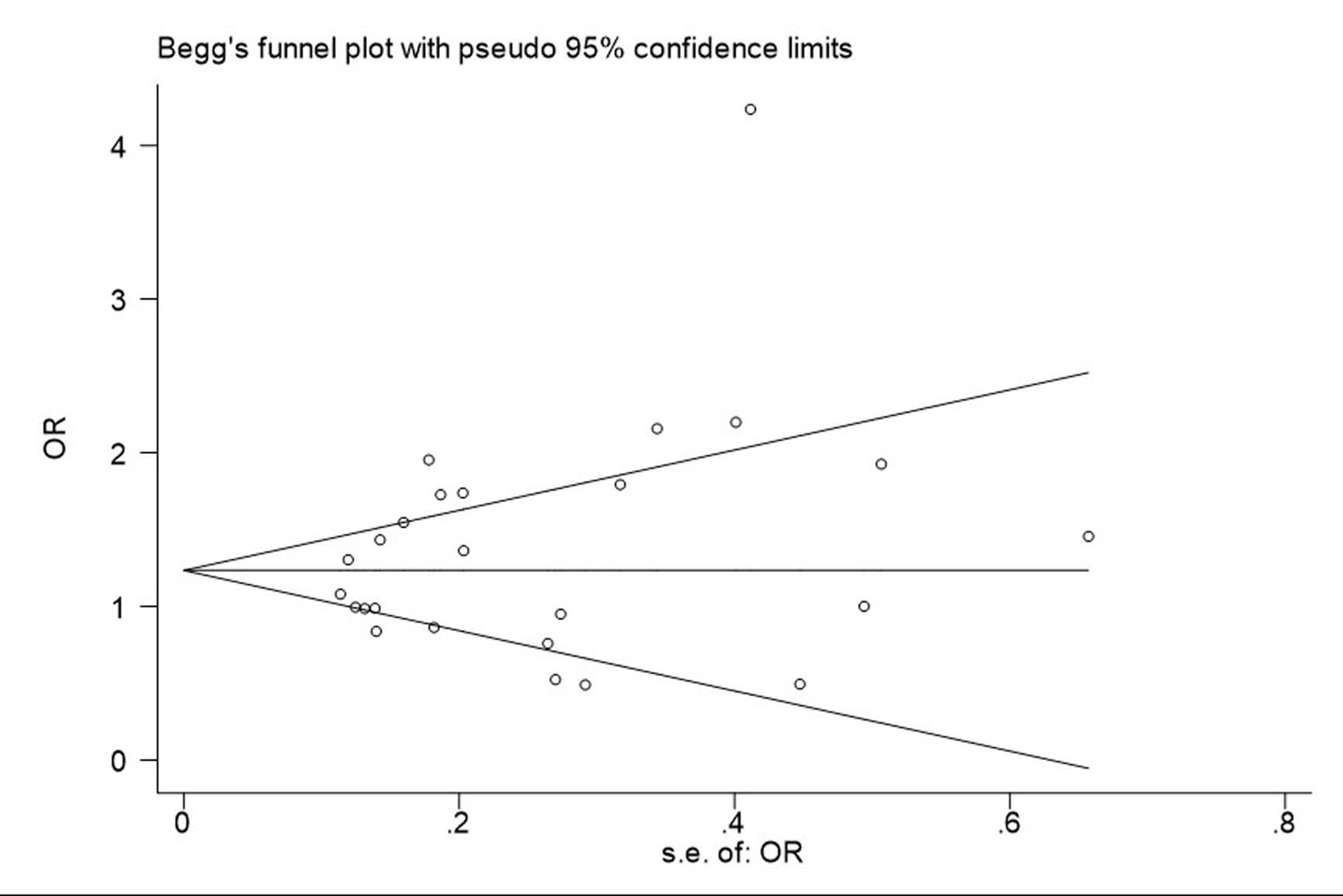

Supplement: Supplementary Figure 1 — The result of publication bias between the allele of TNF-α (G:A) and IS. [file Image_1.TIF]

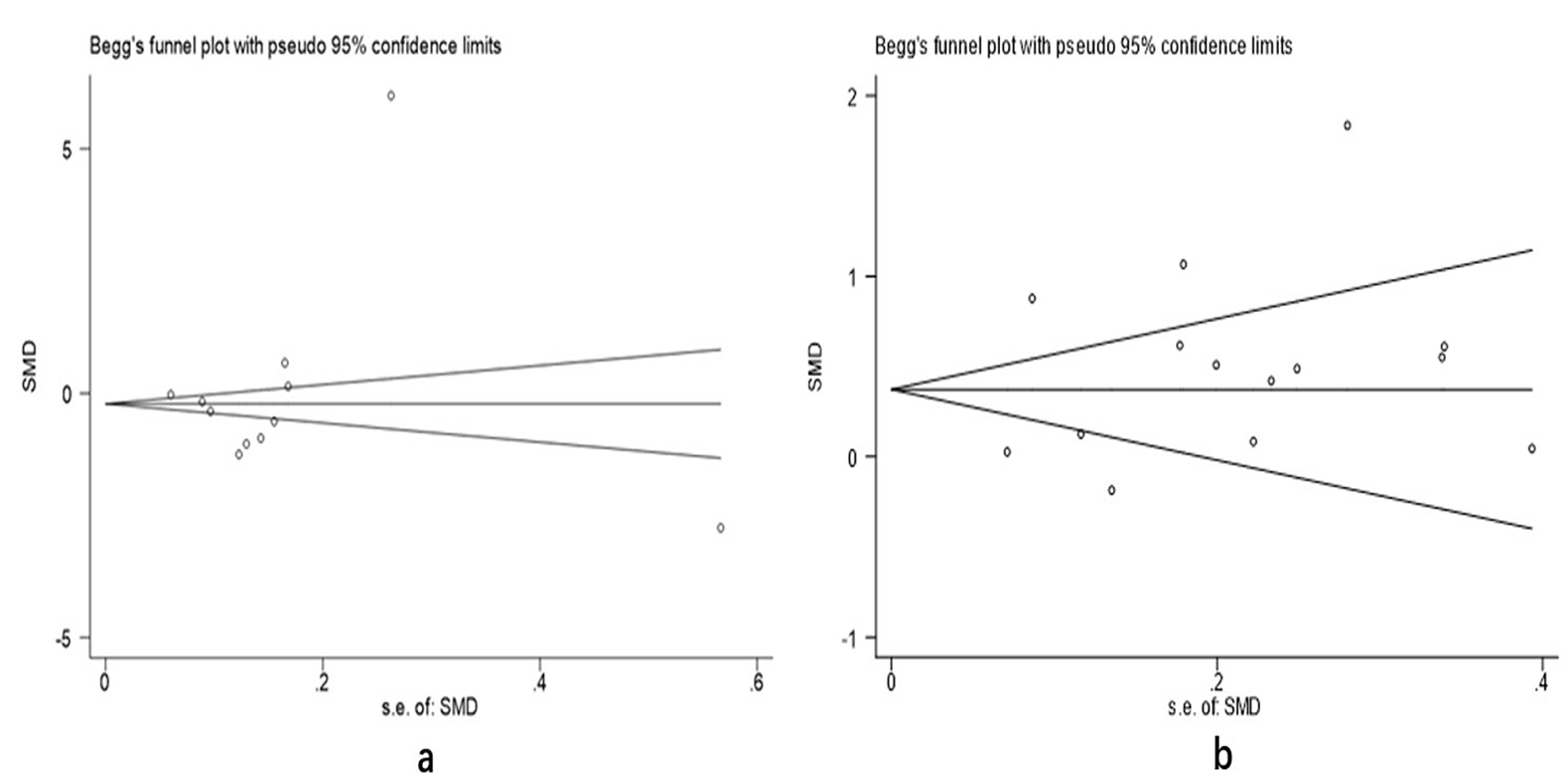

Supplement: Supplementary Figure 2 — The result of publication bias between IGF-1, TNF-α, and IS. (A) The result of publication bias between IGF-1 and IS and (B) the result of publication bias between TNF-α and IS. [file Image_2.TIF]
